# Supplementary material for: Predicting visual function by interpreting a neuronal wiring diagram
Source: Nature. 2024 Oct 2;634(8032):113–23. doi: 10.1038/s41586-024-07953-5 (PMC11446822; doi:10.1038/s41586-024-07953-5)
Supplement: Supplementary file 6 — CRF and ERF predictions for individual TmY4 and TmY9 cells. Analogous to Supplementary Data 3, but for TmY target types. Shown are the top four monosynaptic pathways, the strongest pathway passing through each of the top ten intermediary types (ranking from Extended Data Fig. 7), and the trisynaptic pathway Tm1–TmY–Dm3–TmY (see the section entitled Prediction of spatial normalization). [file 41586_2024_7953_MOESM6_ESM.zip › DataS4/TmY9qΓèÑ/720575940627036478.pdf]

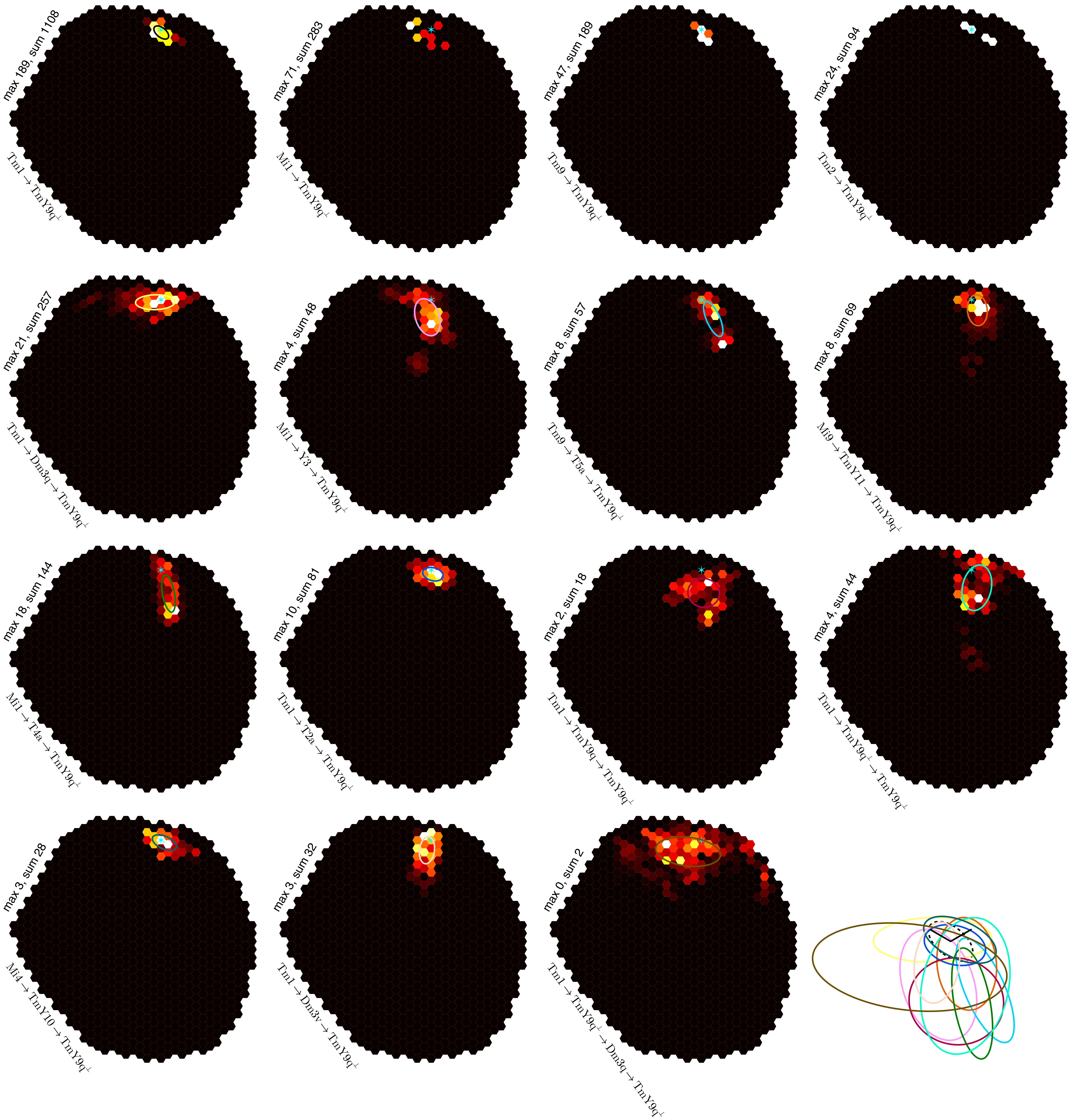
max 189, sum 1108
Tm1 → TmY9q⊥
max 71, sum 283
Mi1 → TmY9q⊥
max 47, sum 189
Tm9 → TmY9q⊥
max 24, sum 94
Tm2 → TmY9q⊥
max 21, sum 257
Tm1 → Dm3q → TmY9q⊥
max 4, sum 48
Mi1 → Y3 → TmY9q⊥
max 8, sum 57
Tm9 → T5a → TmY9q⊥
max 8, sum 69
Mi9 → TmY11 → TmY9q⊥
max 18, sum 144
Mi1 → T4a → TmY9q⊥
max 10, sum 81
Tm1 → T2a → TmY9q⊥
max 2, sum 18
Tm1 → TmY9q → TmY9q⊥
max 4, sum 44
Tm1 → TmY9q⊥ → TmY9q⊥
max 3, sum 28
Mi4 → TmY10 → TmY9q⊥
max 3, sum 32
Tm1 → Dm3v → TmY9q⊥
max 0, sum 2
Tm1 → TmY9q⊥ → Dm3q → TmY9q⊥
